# Up-Regulation of RACK1 by TGF-β1 Promotes Hepatic Fibrosis in Mice

**DOI:** 10.1371/journal.pone.0060115

**Published:** 2013-03-29

**Authors:** Dongwei Jia, Fangfang Duan, Peike Peng, Linlin Sun, Xiaojuan Liu, Lan Wang, Weicheng Wu, Yuanyuan Ruan, Jianxin Gu

**Affiliations:** 1 Department of Biochemistry and Molecular Biology, Shanghai Medical College, Fudan University, Shanghai, P.R. China; 2 Institute of Biomedical Science, Fudan University, Shanghai, P.R. China; Bambino Gesu' Children Hospital, Italy

## Abstract

Liver fibrosis represents the consequences of a sustained wound healing response to chronic liver injury, and activation of quiescent hepatic stellate cells (HSCs) into a myofibroblast-like phenotype is considered as the central event of liver fibrosis. RACK1, the receptor for activated C-kinase 1, is a classical scaffold protein implicated in numerous signaling pathways and cellular processes; however, the role of RACK1 in liver fibrosis is little defined. Herein, we report that RACK1 is up-regulated in activated HSCs in transforming growth factor beta 1 (TGF-β1)-dependent manner both *in vitro* and *in vivo*, and TGF-β1 stimulates the expression of RACK1 through NF-κB signaling. Moreover, RACK1 promotes TGF-β1 and platelet-derived growth factor (PDGF)-mediated activation of pro-fibrogenic pathways as well as the differentiation, proliferation and migration of HSCs. Depletion of RACK1 suppresses the progression of TAA-induced liver fibrosis *in vivo*. In addition, the expression of RACK1 in fibrogenic cells also positively correlates well with the stage of liver fibrosis in clinical cases. Our results suggest RACK1 as a downstream target gene of TGF-β1 involved in the modulation of liver fibrosis progression *in vitro* and *in vivo*, and propose a strategy to target RACK1 for liver fibrosis treatment.

## Introduction

Liver fibrosis represents the consequences of a sustained wound healing response to various chronic liver injuries including viral, autoimmune, drug-induced, cholestatic and metabolic diseases [1]. In response to injury, HSCs undergo a well-characterized activation process during which they lose their characteristic vitamin A and lipid stores, and obtain a myofibroblastic phenotype [2]. This process is associated with the increased expression of contractile filaments such as α-smooth muscle actin (α-SMA) and production of extracellular matrix (ECM), and a large amount of production of pro-fibrogenic factors such as cytokines and reactive oxygen species (ROS) [3]. These cytokines, including interferons, interleukins, tumor necrosis factors (TNF), growth factors (e.g. TGF, PDGF) and chemokines, are released from hepatocytes, Kupffer cells, stellate cells, epithelial cells, endothelial cells and so on, and play critical roles in liver fibrosis through regulating hepatic inflammation, cell necrosis and apoptosis, and ECM production [4].

TGF-β is a cytokine/growth factor with immunosuppressive, anti-inflammatory, and pro-fibrotic properties. In the liver, TGF-β1 is the most abundant isoform, and is secreted by immune cells, epithelial cells and stellate cells. Bone marrow-derived and liver resident macrophages (Kupffer cells) are believed to be the major source of TGF-β1 in fibrotic liver, and the activated HSCs are also an important source of TGF-β1 in the liver [4], [5], [6]. During hepatic fibrogenesis, TGF-β1 plays a pivotal role in the progression of liver fibrosis by promoting the transdifferentiation and migration of HSCs [7], [8]. It has been reported that activated HSCs, which are the principal cells to produce type I collagen in the fibrotic liver, contribute to the development of liver fibrosis through autocrine and paracrine loops of TGF-β1-stimulated collagen production [9]. Therefore, TGF-β1 is considered as a major factor in accelerating the progression of liver fibrosis. The classical Smads pathways (receptor-mediated Smads, Smad2/3), MAPKs as well as NF-κB signaling, have been reported to be implicated in the pro-fibrogenic effect of TGF-β1 [10], [11], [12]. In addition to TGF-β1, PDGF, mainly produced by Kupffer cells, is the most potent mitogen for HSCs and also upregulated in the fibrotic liver [13]. PDGF induces the activation of several signaling pathways such as MAPKs and AKT, which have been reported to involved in the modulation of HSCs proliferation and migration during the progression of liver fibrosis [14], [15]

The receptor for activated C-kinase 1 (RACK1) is a member of the tryptophan-aspartate repeat (WD-repeat) family of proteins. RACK1 possesses the capacity for interaction with diverse proteins and plays important roles in a variety of physiological functions. The altered RACK1 expression has been identified in several kinds of human diseases, including brain developmental disorders, heart failure, muscle atrophy, breast cancer and pulmonary adenocarcinomas [16], [17], [18], and our previous research also indicates that RACK1 is frequently up-regulated in hepatocellular carcinoma and promotes its chemo-resistance and growth [19]. However, the role of RACK1 in liver fibrosis is little defined. In this study, we demonstrate that the expression of RACK1 is increased in activated HSC in TGF-β1 -dependent manner, and RACK1 promotes the initiation and progression of liver fibrosis by enhancing TGF-β1 and PDGF-induced differentiation, migration and proliferation of HSCs. Our results suggest RACK1 as a downstream target gene of TGF-β1 involved in the modulation of liver fibrogenesis, and propose a strategy to target RACK1 for liver fibrosis treatment.

## Materials and Methods

### Ethics Statement

The use of human tissue samples and clinical data was approved by the ethics committee of Fudan University. The individual in this manuscript has given written informed consent (as outlined in PLOS consent form) to publish these case details. Animal experiments were performed according to the criteria outlined in the Guide for the Care and Use of Laboratory Animals, prepared by the National Academy of Sciences and published by the National Institutes of Health, and also approved by the ethics committee of Fudan University. The surgery of liver perfusion was performed under sodium pentobarbital anesthesia, and all efforts were made to minimize suffering.

### Mouse model of liver fibrosis

Wild-type and *Tgfb1+/−* mice of C57BL/6J background were used for inducing liver fibrosis. The wild-type C57BL/6J mice were obtained from SLAC Laboratory Animal Corp (Shanghai, China), and *Tgfb1+/−* mice were purchased from JACKSON laboratory. All mice were maintained at 25°C with a 12 h dark/light cycle. Liver fibrosis was induced by intraperitoneal injection of thioacetamide (TAA) (Sigma) at 0.2 mg/g body weight 3 times each week for 8 weeks. The control mice were received intraperitoneal injection of vehicle (distilled water). For the shRACK1 *in vivo* experiment, RACK1 shRNA lentiviral particles were delivered using the tail vein injection method [20]. Meanwhile, the mice were received TAA or vehicle treatment for 8 weeks. Samples of mouse livers were fixed in formaldehyde or immediately frozen in liquid nitrogen.

### Patients and tissue samples

Forty-six liver biopsy samples were collected from patients with liver fibrosis at Zhongshan Hospital of Fudan University (Shanghai, China). The stage of liver fibrosis is according to previous report [21].

### Primary cells and cell line

Primary hepatic stellate cells were isolated from mice using a modification of the methods of Geerts et al [22]. Briefly, the inferior vena cava was ligated and the portal vein was dissected. The liver was then perfused with Liver Perfusion Medium (GIBCO) followed by pronase E (Sigma Aldrich) and collagenase IV solution treatment. Digested liver tissue was filtered through nylon mesh and centrifugation to remove the parenchymal cells. The nonparenchymal supernatant was subjected to HSC separation by density gradient centrifugation. The purity was assessed by the autofluorescence of vitamin A droplets, and was between 90% and 95%. Primary hepatocytes were isolated by two-step collagenase perfusion [23]. Hepatocytes were filtered through 100 µM filter gaze, and then centrifuged to remove suspension. The HSC-T6 cell line was purchased from XiangYa Central Experiment Laboratory (Changsha, Hunan province, China). All primary cells and cell line were cultured in Dulbecco's modified Eagle's medium (Sigma Aldrich, USA) supplemented with 10% fetal bovine serum (Gibco) at 37°C in a humidified atmosphere with 5% CO2.

### Antibodies and reagents

Recombinant human TGF-β1 and mouse anti-TGF-β1 antibody were obtained from R&D system. SB203580 and SB431542 were purchased from CalBiochem. Rabbit anti-phospho-JNK, -JNK, -phospho-p38, -p38, -phospho-Smad2, -phospho-Smad3, -Smad2/3, -phospho-IKKα/β, -IKKα, -IKKβ, mouse anti-p65 antibodies and U0126 were purchased from Cell Signaling Technology. Rabbit anti-p50 antibody was obtained from BioVision and Santa Cruz Biotechnology. Rabbit anti-ERK1/2, -ERK, mouse anti-GAPDH, -phospho-I-κBα, -I-κBα, -RACK1, and goat anti-Col1A1 antibodies were obtained from Santa Cruz Biotechnology. Rabbit anti-RACK1 antibody was purchased from Abcam. Mouse anti-α-SMA antibody and PDTC were from Sigma Aldrich.

### Plasmid construction, transfection and RNA interference

The NF-κB binding element of *GNB2L1* promoter was subcloned into pGL3-Basic Vector. Transfection was performed using Lipo2000 (Invitrogen) according to manufacturer's instructions. The mouse RACK1 shRNA lentiviral particles were purchased from Santa Cruz Biotechnology.

### Western-blot

Western-blot analysis was carried out according to our previous report [19].

### Immunofluorenscence analysis

For immunofluorescence staining of RACK1 and α-SMA, 8 µm cryostat sections were fixed in ice-cold acetone for 10 min, blocked with 10% goat serum for 2 h at room temperature and incubated with antibodies for RACK1 and α-SMA overnight at 4°C. Immunofluorescence staining was examined using the confocal laser scanning microscopy (Leica Microsystems Heidelberg GmbH, Germany).

### Histological analysis

For HE staining, the sections were stained with hematoxylin, rinsed with water, and stained with eosin, dehydrated and mounted. For Sirius red staining, sections were stained with Sirius red for 25 min, rinsed with 100% ethanol, dehydrated and mounted. For immunohistochemical staining, sections were soaked in 0.3% hydrogen peroxide, and incubated with primary antibody overnight at 4°C. All slides were processed using the peroxidase-antiperoxidase method (Dako, Hamburg, Germany).

For the scoring of RACK1 in clinical samples, double stained sections of RACK1 and α-SMA were interpreted under microscopic fields of 200 or 400-fold magnification. RACK1 scoring was based on a semi-quantitative method according to the intensity and percentage of staining in α-SMA positive cells, which was described previously [19]. The intensity of staining was scored on a scale of 0 to 3, in which 0 = negative staining, 1 = weakly positive staining, 2 = moderately positive staining, and 3 = strongly positive staining. The percentage of staining was estimated on a scale of 0 to 4, in which 0 = none, 1 = 1–25% positive staining in α-SMA positive cells. 2 = 26–50% positive staining; 3 = 51–75% positive staining; and 4 = 76–100% positive staining. The immunohistochemical score (IS) was calculated through multiplying the intensity score by the percentage score. Samples with IS between 0 and 1 were classified as Score 0, samples with IS between 2 and 4 were Score 1, samples with IS between 5 and 8 were Score 2, and samples with IS between 9 and 12 were Score 3. No samples from the 46 cases was grouped as Score 0. All of the sections were evaluated in a blinded manner without knowledge of the clinical and pathological parameters of the patients.

### Real-time PCR analysis

Total RNA was extracted with TRIZOL (Invitrogen) according to manufacturer's instructions. Quality of the total RNA was detected by spectrophotometer (Pharmacia Biotech RNA/DNA Calculator). About 3 µg total RNA from each sample was used to perform reverse-transcribed by using RNA PCR Kit AMV (Takara). Real-time PCR was performed using SYBR Green Premix Ex Taq Ver. 3.0 (Takara) and detected by StepOne plus. The following primers were used: GAPDH Forward: GAGCGAGACCCCACTAACAT; Reverse: TCTCCATGGTGGTGAAGACA; RACK1 Forward: GGATCTCAATGAAGGCAAGC; Reverse: TTGCTGCTGGTGCTGATAAC

### Luciferase activity assay

For luciferase activity assay, cell extracts were prepared and luciferase activities were detected according to the manufacture's instructions (Promega) using a Lumat LB9507 luminometer. All assays were performed in triplicate.

### Chromatin immunoprecipitation assay (ChIP)

ChIP was performed using a commercial kit according to the manufacturer's instructions (Active Motif-ChIP-IT™ Express). Proteins cross-linked to DNA were immunoprecipitated with antibodies against p65, p50 or control IgG. The purified DNA was amplified by the promoter-specific primers. RACK1 Forward: ACTTGAGACCTACTCTCCCAG and Reverse: GGATAGCTCAGAGAGCCAAGC, and PCR products were separated by gel electrophoresis on 2% agarose gel and visualized.

### Co-immunoprecipitation

Primary HSCs were treated with or without TGF-β1, washed with ice-cold phosphate-buffered saline (PBS) and solubilized with co-immunoprecipitation (co-IP) buffer. Cell lysates were rotated with relevant antibody and Co-IP was performed as before [19].

### Migration assay

Migration assay was performed according to a previous report [8]. Briefly, the membranes with 8-µm pores were coated with type IV collagen on the upper side and with type I collagen on the lower side. Cells were added into the upper chamber. The number of infiltrating cells was counted from five visions and the experiments were repeated three times.

### Cell proliferation assay

Cell proliferation was determined using Cell Counting Kit-8 (CCK-8) (Beyotime Inst. Biotech, China) according to manufacture's instructions. Briefly, cells were seeded in a 96-well plate and treated with or without PDGF, then incubated with WST-8 dye at 37°C. And the absorbance was determined at 450 nm using a Universal Microplate Reader (Bio-Tek Instrument Inc.). All assays were performed in triplicate.

### Statistical analysis

Results were presented as mean ± S.D. Statistical analysis was performed using One-way ANOVA analysis. Statistical significance was determined at the level of p<0.05.

## Results

### RACK1 is up-regulated in activated HSCs in vivo and in vitro

**Figure 1:**
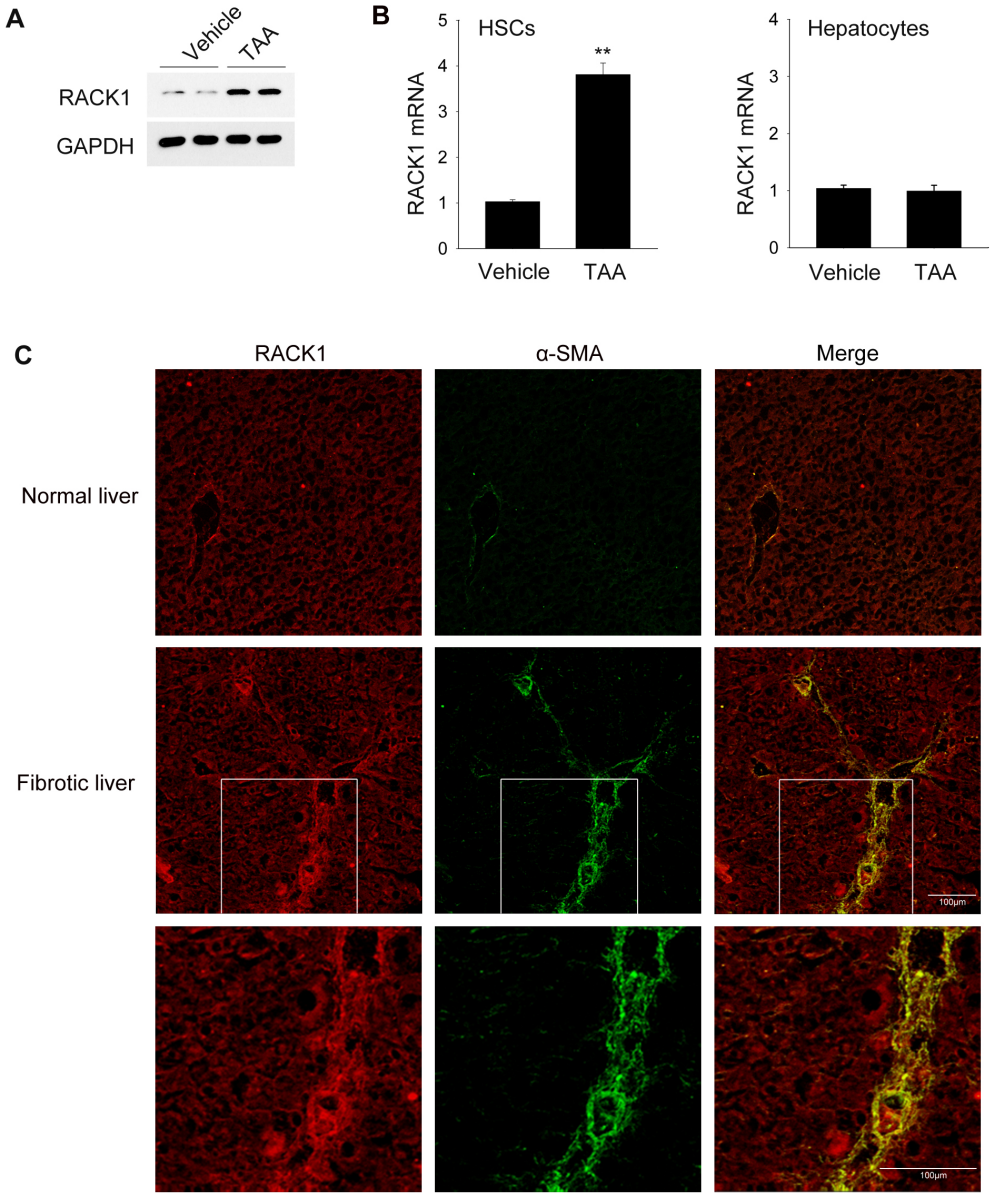
RACK1 is up-regulated in activated HSCs *in vivo*. The C57BL/6 mice were treated with vehicle or TAA for 8 weeks. (A) Freshly isolated HSCs from vehicle or TAA-treated mice were collected, and cell lysates were applied to western-blot analysis to detect RACK1 expression. (B) Freshly isolated HSCs or hepatocytes from vehicle or TAA-treated mice (n = 6) were applied to qRT-PCR analysis to assess RACK1 transcripts with GAPDH mRNA as the internal control. **, p<0.01. (C) Immunofluorescence staining against RACK1 (red) and α-SMA (green) was performed using frozen liver sections from vehicle or TAA-treated mice.

RACK1 is a classical scaffold protein ubiquitously expressed in the tissues of higher mammals and humans, and plays critical roles in the regulation of various signaling pathways and numerous aspects of cellular functions. To understand the role of RACK1 in the development of liver fibrosis, we examined the expression pattern of RACK1 in HSCs using the mouse model of TAA-induced liver fibrosis. As shown in Figure 1A and B, RACK1 expression was significantly up-regulated in activated HSCs after TAA treatment, but not in hepatocytes. Immunofluorescence analysis also revealed that the number of RACK1 and α-SMA (a marker for activated HSCs) double-positive cells was markedly increased in TAA-treated liver compared to vehicle control (Figure 1C). These results suggest that RACK1 is up-regulated in activated HSCs during the development of liver fibrosis *in vivo*.

**Figure 2:**
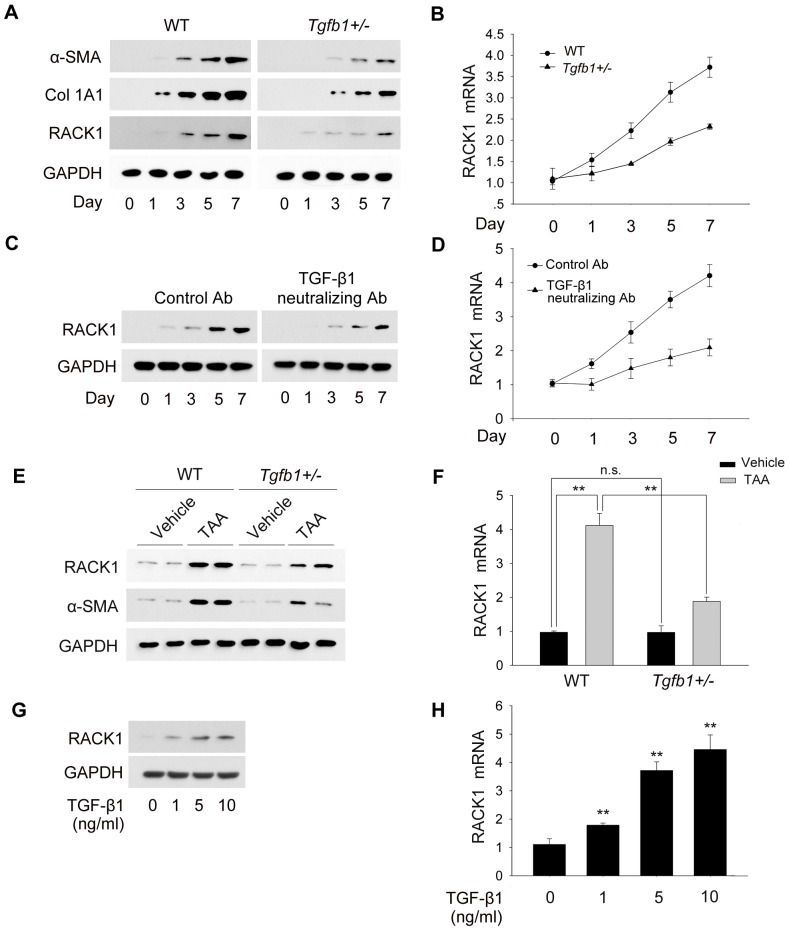
TGF-β1 is involved in the up-regulation of RACK1 in activated HSCs. (A, B) HSCs were isolated from wild-type or *Tgfb1^+/−^* mice, cultured for indicated time periods, and subjected to western blot (A) and qRT-PCR (B). (C, D) Freshly isolated HSCs were treated with neutralizing TGF-β1 antibody for indicated time periods, and subjected to western blot (C) and qRT-PCR (D) analysis. (E, F) Wild-type or *Tgfb1^+/−^* mice were treated with vehicle or TAA for 8 weeks. Freshly isolated HSCs were applied to western-blot and qRT-PCR analysis to detect relative RACK1 and α-SMA expression. (G, H) Freshly isolated HSCs after adherence were treated with TGF-β1 at varying concentrations for 48 h (G) or 6 h (H), harvested and subjected to western blot (G) and qRT-PCR (H) analysis to detect RACK1 expression. **, p<0.01.

It has been reported when cultured on plastic, freshly isolated quiescent HSCs undergo spontaneous fibrogenic activation to myofibroblast-like cells [24]. To further confirm whether RACK1 was up-regulated in activated HSCs, we next detected the expression profile of RACK1 in primary HSCs *in vitro*. With the progression of HSCs auto-differentiation, the protein and mRNA levels of RACK1 were coordinately increased (Figure 2A, left panel and 2B). Taken together, these results suggest that RACK1 expression is increased in activated HSCs *in vivo* and *in vitro*.

### TGF-β1 is involved in the up-regulation of RACK1 in activated HSCs

Cytokines regulating the inflammatory response to injury play critical roles in the modulation of hepatic fibrogenesis, and TGF-β1 is considered as the most pro-fibrogenic cytokine and mediates the activation of HSCs and ECM production [25], [26]. To explore whether TGF-β1 was required for HSCs activation, we next examined the expression of α-SMA and collagen in *Tgfb1* heterozygous HSCs. As shown in Figure 2A, the up-regulation of α-SMA and collagen in *Tgfb1* heterozygous HSCs was suppressed during the auto-differentiation *in vitro*. Moreover, TGF-β1-deficiency remarkably delayed the protrusions extending from the cell body as well as the recession of the vitamin A lipid droplet (Figure S1). These results suggest that TGF-β1 is required for auto-activation of HSCs *in vitro*.

To determine TGF-β1 is involved in the regulation of RACK1 expression, we also examined the expression profile of RACK1 in HSCs isolated from *Tgfb1* heterozygous mice *in vitro*. As shown in Figure 2A and 2B, TGF-β1-deficiency impaired the up-regulation of RACK1 in primary HSCs during the auto-differentiation in *vitro*. Moreover, TGF-β1 neutralizing antibody efficiently inhibited the elevation of RACK1 protein and mRNA levels during the auto-activation of HSCs,suggesting that TGF-β1-induced RACK1 expression is in the autocrine manner (Figure 2C and D). We also examined the expression pattern of RACK1 in HSCs from *Tgfb1* heterozygous mice in vivo. As shown in Figure 2E and F, the increase in RACK1 protein and mRNA levels upon TAA treatment was remarkably attenuated in *Tgfb1* heterozygous HSCs in vivo, by comparing with that in wild-type HSCs. We next examined whether TGF-β1 treatment induced RACK1 expression in primary HSCs. As shown in Figure 2G and H, administration of recombinant TGF-β1 induced the elevation of RACK1 protein and mRNA levels both in dose-dependent manner. These data suggest that TGF-β1 is involved in regulation of RACK1 expression in HSCs *in vitro* and *in vivo*

### TGF-β1 promotes the expression of RACK1 in NF-κB-dependent manner

**Figure 3:**
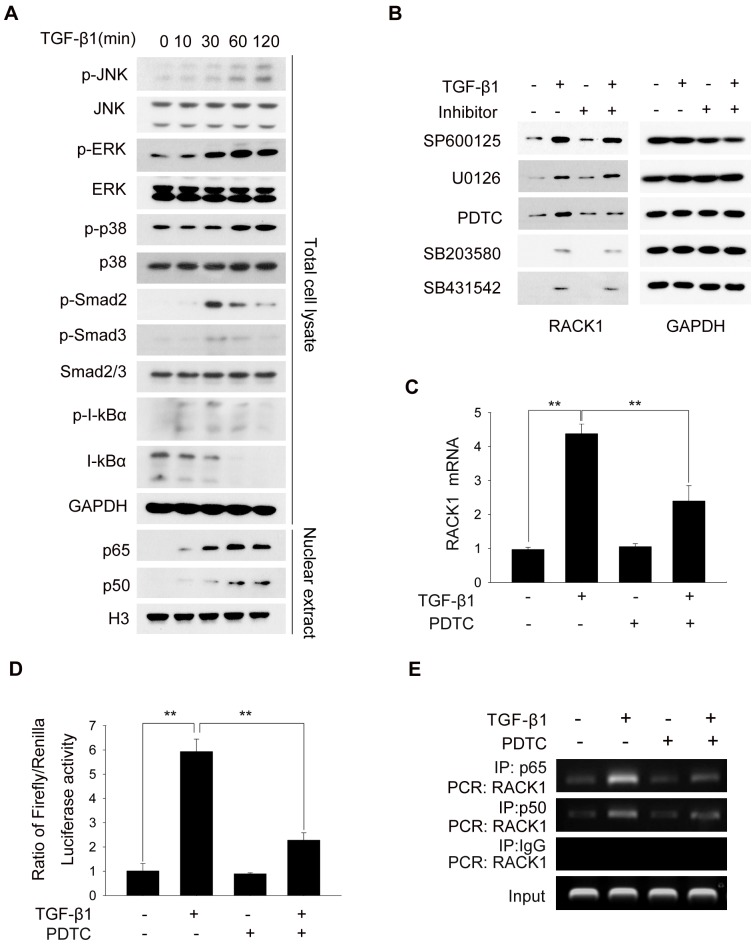
TGF-β1 promotes the expression of RACK1 in NF-κB-dependent manner. (A) Primary mouse HSCs were serum-starved for 6 h, and treated with TGF-β1 (10 ng/ml) for indicated times. Total cell lysates or nuclear extracts were subjected to western blot analysis. (B) Primary mouse HSCs were treated with TGF-β1 (10 ng/ml) for 48 h, along with vehicle, SP600125, U0126, PDTC, SB203580 or SB431542. (C) Primary mouse HSCs were stimulated with TGF-β1 for 6 h, along with or without PDTC, followed by qRT-PCR analysis. (D) The pGL3 vector carrying the NF-κB binding element of GNB2L1 promoter was transfected into HSC-T6 cells. 24 h later, cells were treated with TGF-β1 and/or PDTC for another 24 h for luciferase activity assay. (E) Primary mouse HSCs were treated with TGF-β1 and/or PDTC for 6 h, followed by ChIP assay. The data shown in (E) are representative of three independent experiments. **, p<0.01.

To explore the underlying mechanism how TGF-β1 modulated the expression of RACK1, we first examined TGF-β1-mediated signaling pathways in primary HSCs. As shown in Figure 3A, treatment of TGF-β1 triggered the activation of JNK, ERK, p38 and Smad2/3 signaling. Moreover, TGF-β1 also induced the phosphorylation and degradation of IκBα, as well as the translocation of p65 and p50 NF-κB subunits into nucleus.

Previous reports have suggested that NF-κB and JNK pathways regulate RACK1 transcription and expression [24], [27], [28]. Therefore, we next determined whether NF-κB and/or JNK signaling mediated the up-regulation of RACK1 induced by TGF-β1. As shown in Figure 3B and C, treatment of PDTC, an inhibitor of NF-κB, but not the JNK inhibitor SP600125, suppressed TGF-β1-induced up-regulation of RACK1 both at the protein and mRNA levels. Moreover, inhibition of ERK, p38 and Smad2/3 signaling pathway showed little effect on TGF-β1-induced RACK1 expression.

To further confirm whether NF-κB was involved in TGF-β1-mediated RACK1 up-regulation, HSC-T6 cells were transfected with pGL3-Basic vector carrying NF-κB binding element of *GNB2L1* promoter, and luciferase gene reporter assay was carried out to examine the effect of TGF-β1 on the activity of the NF-κB binding element. Results demonstrated that TGF-β1 treatment induced a remarkable increase in the luciferase activity in NF-κB-dependent manner (Figure 3D). ChIP assay also revealed that administration of TGF-β1 enhanced the binding of p65 and p50 NF-κB subunits to the promoter of *GNB2L1* in HSCs, and PDTC attenuates the interaction between NF-κB subunits and *GNB2L1* promoter (Figure 3E). These results imply that TGF-β1 promotes the expression of RACK1 in NF-κB-dependent manner.

### RACK1 is involved in the regulation of TGF-β1-mediated signaling pathways in HSCs

**Figure 4:**
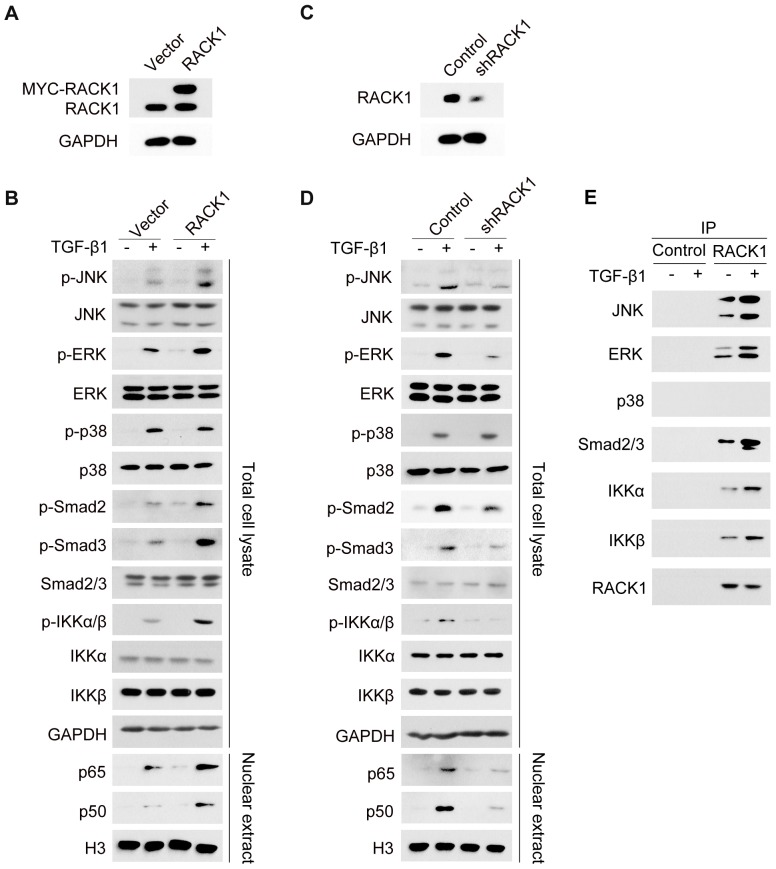
RACK1 is involved in the regulation of TGF-β1-mediated signaling pathways in HSCs. (A, B) Primary mouse HSCs were incubated with lentivirus expressing Myc-RACK1 or control for 96 h. Then cells were treated with TGF-β1 (10 ng/ml) and subjected to western blot analysis. (C, D) Primary mouse HSCs were incubated with lentiviral particles expressing shRNA targeting RACK1 or non-target control for 96 h. Then cells were treated with TGF-β1 (10 ng/ml) and subjected to western blot analysis. (E) Primary HSCs were treated with TGF-β1. Cell lysates were subjected to immuoprecipitation with anti-RACK1 antibody, followed by western blot with related antibodies.

Several reports have demonstrated that RACK1 interacts with the components of numerous signaling pathways [29], [30], [31], and we next examined the effect of RACK1 on TGF-β1-induced pro-fibrogenic signaling in HSCs. As shown in Figure 4A and B, overexpression of RACK1 promoted TGF-β1-induced phosphorylation of JNK, ERK, Smad2/3 and IKKα/β as well as translocation of NF-κB subunits p65 and p50 into nucleus, while inhibition of RACK1 expression using specific shRNA attenuated TGF-β1-induced JNK, ERK, Smad2/3, IKKα/β and NF-κB activation (Figure 4C and D). However, RACK1 showed little effect on TGF-β1-induced p38 activation (Figure 4A–D).

RACK1 is a classical scaffold protein possessing the capacity for interaction with diverse proteins and regulation of multiple signaling pathways. To understand the role of RACK1 in TGF-β1-induced early signaling, we next examined the interaction between RACK1 and the early signaling components of TGF-β1 pathway. As shown in Figure 4E, RACK1 associated with JNK, ERK, Smad2/3 as well as IKKα/β, and their interactions were further enhanced upon TGF-β1 treatment. However, the interaction between RACK1 and p38 was not detected. These results suggest that RACK1 functions as a critical scaffold protein in TGF-β1-induced early signaling.

### RACK1 is involved in the regulation of TGF-β1-mediated differentiation and migration of hepatic stellate cells

**Figure 5:**
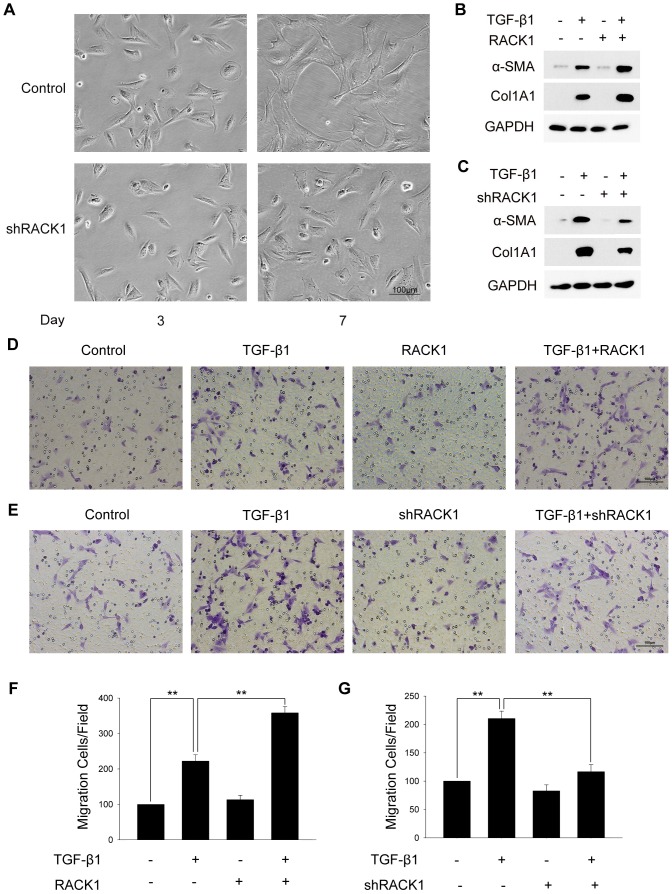
RACK1 is involved in the regulation of TGF-β1-mediated differentiation and migration of HSCs. (A) Fresh isolated primary mouse HSCs were incubated with lentiviral particles expressing shRNA targeting RACK1 or non-target control. The images were captured at the day of 3 and 7 after isolation. Scale bar, 100 µm. (B, C) 96 h after incubation with lentiviral particles, HSCs were stimulated with TGF-β1 (10 ng/ml) for 24 h, and expression of α-SMA and collagen α1 type 1 was analyzed. (D–G) Primary HSCs were incubated with lentivirus as indicated, and treated with or without TGF-β1, followed by transwell assay. In (D) and (E), images are representative of three independent experiments. In (F) and (G), the number of infiltrating cells was counted in five regions selected at random, and the experiments were repeated three times. **, p<0.01.

To understand whether RACK1 was involved in the regulation of HSCs activation, we first examined the effect of RACK1 on the morphological changes of HSCs during the auto-differentiation *in vitro*. As shown in Figure 5A, inhibition of RACK1 expression attenuated the spontaneous spreading of primary HSCs *in vitro*, suggesting that RACK1 is involved in the auto-differentiation of HSCs *in vitro*.

Since TGF-β1 is considered as the most potent pro-fibrogenic cytokine and mediates the HSCs activation and ECM production, we next examined the effect of RACK1 on TGF-β1-induced differentiation of HSCs. As shown in Figure 5B and C, overexpression of RACK1 promoted TGF-β1-induced up-regulation of α-SMA and collagen α1 type 1, while knock-down of RACK1 impaired the regulatory effect of TGF-β1 on α-SMA and collagen expression. We also evaluated the role of RACK1 in TGF-β1-induced HSCs migration. Transwell assay revealed that overexpression of RACK1 promoted TGF-β1-induced HSCs migration from upper to the bottom wells of the chamber, while depletion of RACK1 by shRNA suppressed TGF-β1-mediated migration of HSCs (Figure 5D–G). Taken together, these results suggest that RACK1 is involved in the regulation of HSCs activation and migration induced by TGF-β1.

### RACK1 is involved in the regulation of PDGF-mediated HSCs proliferation and migration

**Figure 6:**
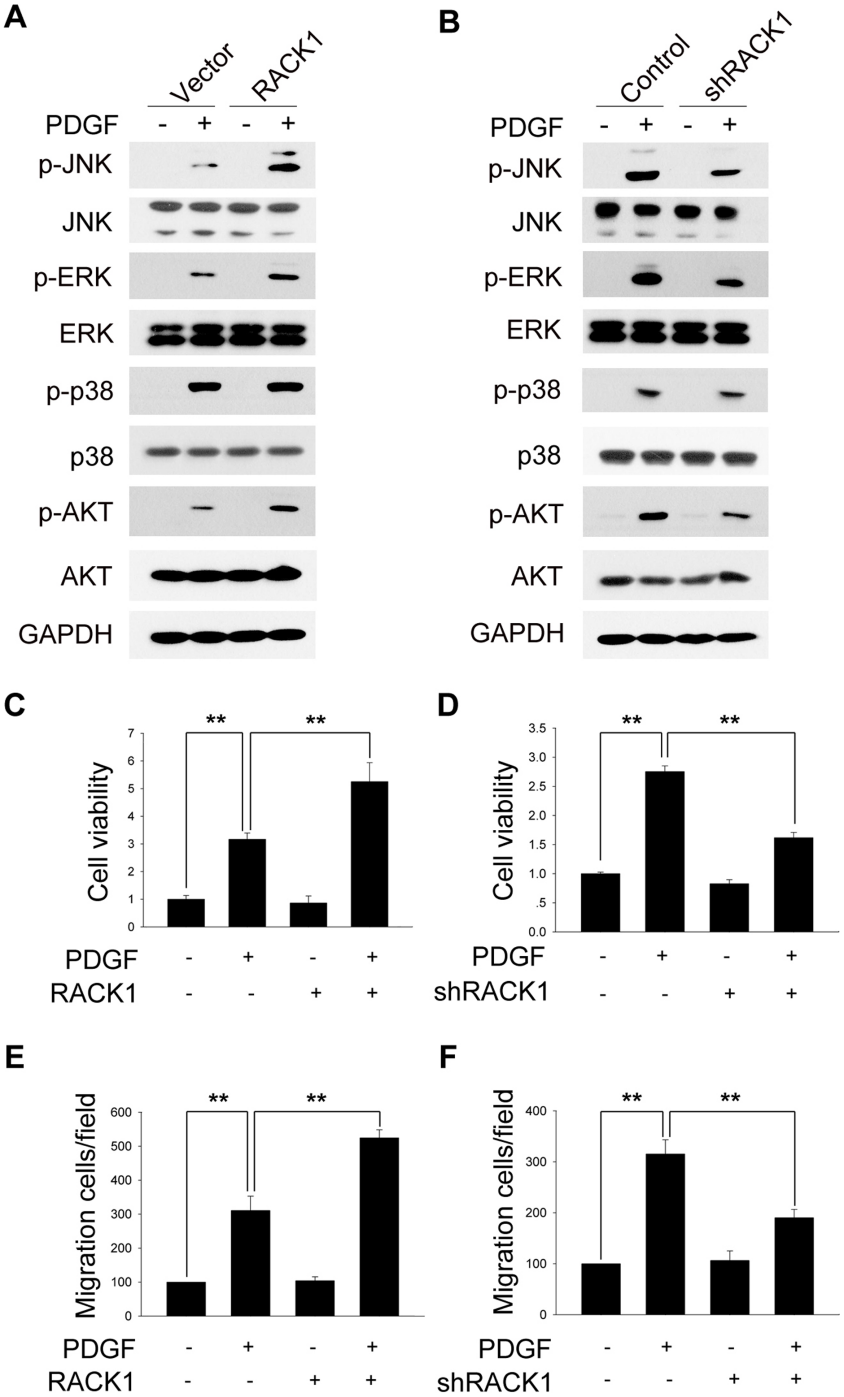
RACK1 is involved in the regulation of the pro-fibrogenic effects of PDGF. (A, B) Primary HSCs were incubated with lentivirus as indicated, starved for 6 h and treated with or without PDGF, followed by western blot analysis. (C, D) Primary HSCs were incubated with lentivirus as indicated, and treated with or without PDGF, followed by CCK8 assay. (E, F) Primary HSCs were treated as in (A, B), followed by migration assay. **, p<0.01.

In addition to TGF-β1, PDGF also plays a critical role in liver fibrogenesis by stimulating the proliferation and migration of HSCs [32]. To understand whether RACK1 also enhanced the profibrogenic effect of PDGF, we first evaluated the role of RACK1 in PDGF-induced activation of profibrogenic signaling pathways. As shown in Figure 6A and B, overexpression of RACK1 promoted PDGF-induced phosphorylation of JNK, ERK, and AKT, while inhibition of RACK1 expression using specific shRNA attenuated PDGF-induced activation of JNK, ERK, and AKT.

We also examined the role of RACK1 in PDGF-induced proliferation and migration of HSCs. As shown in Figure 6C and D, CCK8 assay revealed that overexpression of RACK1 promoted PDGF-induced HSCs proliferation, while depletion of RACK1 suppressed PDGF-mediated HSCs proliferation. Similar effect of RACK1 was also observed in PDGF-induced HSCs migration (Figure 6E and F). Taken together, these results suggest that RACK1 is involved in the regulation of the profibrogenic effect of PDGF.

### RACK1 is involved in the regulation of TAA-induced liver fibrosis in mice

**Figure 7:**
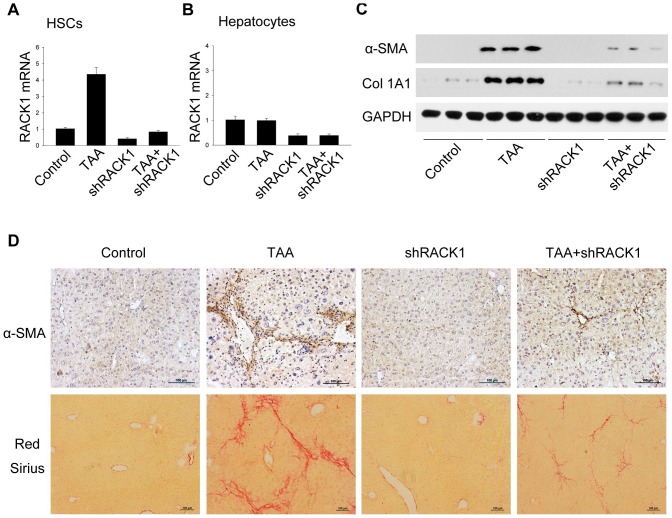
RACK1 is involved in the regulation of TAA-induced liver fibrosis in mice. Mice were treated with lentiviral particles expressing shRNA targeting RACK1 or non-target control, as well as TAA or vehicle control. (A, B) 8 weeks later, HSCs and hepatocytes were isolated and applied to qRT-PCR analysis to detect relative RACK1 expression. (C) Total protein lysates extracted from the livers were applied to western blot for α-SMA and collagen α1 type 1. (D) Liver sections were subjected to immunochemistry for α-SMA and Sirius Red staining. Scale bar, 100 µm.

We next determined the role of RACK1 in TAA-induced liver fibrosis *in vivo*. Delivery of lentivirus carrying shRACK1 effectively suppressed the mRNA level of RACK1 both in HSCs and hepatocytes (Figure 7A and B). Moreover, western blot and histochemical staining also revealed that depletion of RACK1 remarkably attenuated TAA-induced up-regulation of α-SMA and collagen I in liver tissues (Figure 7C and D). Taken together, these results suggest that RACK1 is involved in the regulation of liver fibrogenesis *in vivo*.

### RACK1 expression is positive relative to the stage of liver fibrosis

**Figure 8:**
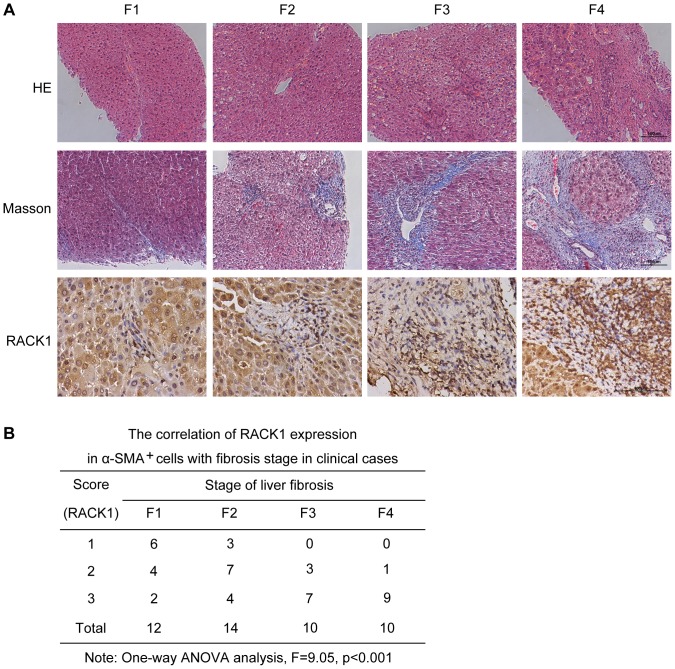
RACK1 expression is positively relative to the stage of liver fibrosis. (A) The liver biopsy samples were collected from patients with different degrees of fibrosis, and applied to HE staining, Masson staining and immunohistochemistry analysis for RACK1. The images shown are representative of each group with different fibrotic stage. (B) The 46 cases were grouped according to the scoring of RACK1, and applied to One-way ANOVA analysis to detect the correlation of RACK1 expression in α-SMA+ cells with the stage of liver fibrosis.

We next evaluated the expression of RACK1 in liver biopsy samples from patients with liver fibrosis. As shown in Figure 8A, the expression of RACK1 in fibrous septum is up-regulated, with the stage of liver fibrosis increases from F1 to F4. To further determine the role of RACK1 expression in fibrogenic cells, we also examined the correlation of RACK1 expression in fibrogenic cells with the fibrosis stage of clinical cases. As shown in Figure 8B, RACK1 expression in α-SMA^+^ cells positively correlated with the stage of liver fibrosis (F = 9.05, p<0.001). Taken together, these results suggest that RACK1 may contribute to the progression of liver fibrosis in clinical cases.

## Discussion

Liver fibrosis is the excessive accumulation of extracellular matrix proteins including collagen that occurs in most types of chronic liver diseases [13]. Advanced liver fibrosis results in cirrhosis, which is the most common non-neoplastic cause of death among hepatobiliary and digestive diseases, accounting for approximately 30000 deaths per year in the US [1]. In this study, we report that the expression of RACK1 is increased in activated HSCs in TGF-β1-dependent manner, and up-regulation of RACK1 induces TGF-β1 and PDGF-mediated pro-fibrogenic pathways as well as the differentiation, migration and proliferation of HSCs.

Activation of quiescent hepatic stellate cells (HSCs) into a myofibroblast-like phenotype is supposed to be the central event of liver fibrosis, and TGF-β1 is considered as a potent pro-fibrogenic cytokine responsible for HSC activation and migration. It has been reported that TGF-β1-deficient mice strongly attenuate the development of liver fibrosis, while overexpression of TGF-β1 in transgenic mice resulted in the acceleration of liver fibrosis progression [6]. TGF-β1 stimulates collagen gene transcription, suppresses MMPs expression and modulates the expression of integrins [33]. In our study, we also identified RACK1 as a novel downstream target of TGF-β1 in liver fibrosis through activating NF-κB. Though there have been reports that NF-κB and c-Jun might both modulate the transcription of RACK1 [27], [28], our results indicated that TGF-β1 mediates the up-regulation of RACK1 in NF-κB-dependent and JNK-independent manners in HSCs, suggesting that the regulation of RACK1 transcription may vary in different types of cells (Figure 3). Moreover, in addition to TGF-β1, there are also several other fibrogenic and inflammatory cytokines that activate macrophages and HSCs, such as IL-6, IL-1β, TNF-α and PDGF. However, we found that administration of recombinant IL-6, IL-1β, TNF-α or PDGF showed little effect on RACK1 expression in HSCs, suggesting that the regulatory effect of TGF-β1 on RACK1 expression is specific in HSCs (data not shown).

RACK1 is a classical scaffold protein involved in the regulation of multiple signaling pathways and cellular functions. Though RACK1 has been reported to be implicated in the development of several kinds of diseases, the role of RACK1 in liver fibrosis is little understood. A previous research implied that RACK1 associated with ADAM12 and promoted its translocation to membrane in PKC-dependent manner in HSCs, suggesting that RACK1 might contribute to the development of liver fibrosis [34]. In our study, we also found that RACK1 functioned as a potent regulator of TGF-β1 and PDGF signaling pathways, and promoted TGF-β1 and PDGF-mediated differentiation, proliferation and migration of HSCs, suggesting an essential role of RACK1 in the regulation of liver fibrogenesis. However, in addition to the HSCs differentiation into a myofibroblast-like phenotype, migration to the injured region and proliferation, there are several other aspects accelerating the initiation and progression of liver fibrosis, such as the survival of HSCs and the imbalance of ECM production and degradation. Since RACK1 has been reported to be involved in the regulation of cellular apoptosis and ADAM12 translation, it is likely that the up-regulation of RACK1 induced by TGF-β1 may contribute to liver fibrosis on other aspects.

Nowadays, mounting clinical evidences suggest that liver fibrosis can regress either by removing the cause of liver injury or treating the underlying diseases; however, there is no licensed anti-fibrotic therapies yet [35]. As the central event of liver fibrosis, activation of HSCs was investigated as the critical therapeutic target. It has been reported that in the animal model, removal of activated HSCs by apoptosis or reversal of hepatic myofibroblats to a quiescent state contributes to the regression of liver fibrosis [2], . Therefore, it is of great potential in developing new agents and strategies to regress liver fibrosis. Our data suggest RACK1 as a novel biomarker in the initiation and progression of liver fibrosis, and propose a strategy to target RACK1 as a potential adjuvant therapy for liver fibrosis treatment.

## Supporting Information

Figure S1 **TGF-β1 is required for the auto-activation of HSCs.** Primary HSCs isolated from wild-type and *Tgfb1+/−* mice were cultured in DMEM supplemented with 10% fetal bovine serum. (A) The images showing the star shaped HSCs were captured at the day of 3 and 7 (scale bar = 100 µm). (B) The images showing vitamin A lipid droplet were captured at the day of 2, 5 and 8 (scale bar = 25 µm).
(TIF)
