# Supplementary figures and images for: Up-Regulation of RACK1 by TGF-β1 Promotes Hepatic Fibrosis in Mice
Source: PLoS One. 2013 Mar 29;8(3):e60115. doi: 10.1371/journal.pone.0060115 (PMC3612079; doi:10.1371/journal.pone.0060115)

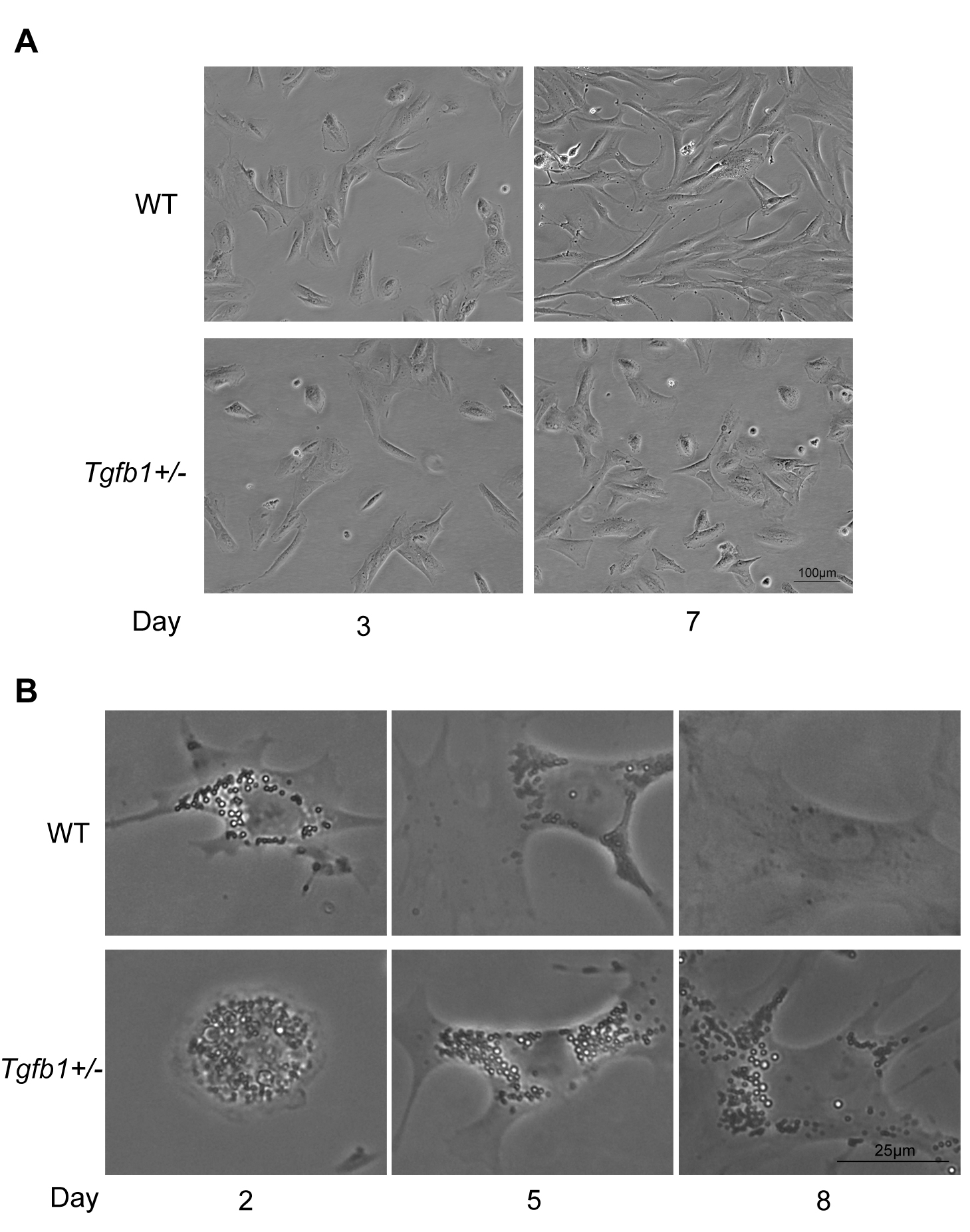

Supplement: Figure S1 — TGF-β1 is required for the auto-activation of HSCs. Primary HSCs isolated from wild-type and Tgfb1+/− mice were cultured in DMEM supplemented with 10% fetal bovine serum. (A) The images showing the star shaped HSCs were captured at the day of 3 and 7 (scale bar = 100 µm). (B) The images showing vitamin A lipid droplet were captured at the day of 2, 5 and 8 (scale bar = 25 µm). (TIF) [file pone.0060115.s001.tif]
